# Integrating landscape genomics and machine learning to analyze the genetic structure and specific molecular markers of Chinese dairy goats (*Capra hircus*)

**DOI:** 10.1186/s40104-026-01473-0

**Published:** 2026-08-06

**Authors:** Jinke Xiong, Mingyu Shang, Jingjing Bao, Tianyi Liu, Peifu Yang, Xintian Sun, Yuting Jiang, Li Zhang

**Affiliations:** State Key Laboratory of Animal Biotech Breeding, Institute of Animal Science, Chinese Academy of Agricultural Sciences (CAAS), Beijing, 100193 China

**Keywords:** Chinese dairy goats, Environmental adaptation, Horn and coat color traits, Machine learning, Population genomics

## Abstract

**Background:**

During long-term artificial selection, China has developed indigenous dairy goat populations with breed-specific traits and environmental adaptability. However, the genetic relationships and genomic characteristics of these populations remain insufficiently studied.

**Results:**

We analyzed 270 whole-genome sequences from 16 goat breeds, including eight Chinese dairy breeds, four introduced dairy breeds, and four other breeds. Population structure analysis revealed significant genetic differentiation among Chinese, European, and African dairy goats. Different dairy goat breeds in China formed geographically distinct subclusters, and multiple gene flow events with introduced breeds were detected. Compared with European and African populations, Chinese dairy goats exhibited higher genetic diversity, lower genomic inbreeding levels, and faster linkage disequilibrium decay. Among five machine learning algorithms evaluated across 10 repeated validations, k-nearest neighbors (KNN) showed the best performance (accuracy = 0.981, AUC = 0.991), and a minimal 134-SNP panel was selected for accurate classification of Chinese dairy goat breeds. Genome–environment association analyses identified candidate genes associated with energy and lipid metabolism, stress response, reproduction, and immune function, with a missense variant in *HDAC10* (chr5:118557145 A > G) showing significant differentiation among Chinese dairy goat populations from different regions. Selection signature and genome-wide association analyses identified candidate genes associated with horn type and coat color, including two missense mutations in *ERG* (chr1:149921788 C > G) and *MITF* (chr22:31686241 T > C).

**Conclusions:**

These findings systematically reveal the genetic structure and genomic characteristics of Chinese dairy goats, providing an important scientific basis for genetic improvement, breed identification, and the conservation of dairy goat genetic resources.

**Supplementary Information:**

The online version contains supplementary material available at 10.1186/s40104-026-01473-0.

## Introduction

As one of the earliest domesticated livestock species, goats have undergone long-term selection for economically important traits [1]. Specifically, dairy goats have been selectively bred as a specialized production type for high milk yield [2, 3]. Since the late nineteenth century, several excellent dairy goat breeds have been introduced worldwide, accelerating the development of the dairy goat industry and facilitating the formation of locally adapted dairy goat breeds in many regions. China is a major goat-producing country with more than 90 breeds classified into diverse production types, including cashmere, meat, fiber, milk, and leather goats. In the early twentieth century, Saanen, Toggenburg, and Nubian goats were introduced into different regions of China, facilitating the development of the Chinese dairy goat industry [4]. Based on these introduced breeds, several dairy goat breeds with high milk yield and local adaptation have been developed in China, including Laoshan, Wendeng, Ya'an, and Guanzhong dairy goats.

Previous studies have used whole-genome resequencing data to investigate the genetic relationships between Guanzhong and several introduced dairy goat breeds [5], and further compared genomic differences between introduced breeds and the Xinong Saanen, Laoshan, and Guanzhong dairy goats [6–8]. However, these studies examined only a few breeds, and research on Chinese dairy goat populations remains incomplete. In recent years, the rapid expansion of the goat milk industry has increased the frequency of breed introduction and crossbreeding across regions, leading to greater breed admixture and complicating breed identification in breeding stock. Machine learning approaches have been applied to breed classification and informative SNP selection in pigs [9], chickens [10], and cattle [11], but their application in Chinese dairy goats remains limited. Therefore, systematic analysis of the genetic structure and diversity of Chinese dairy goats is essential for clarifying breed relationships and supporting reliable molecular breed identification.

The development of Chinese dairy goat populations has been shaped by both natural and artificial selection. Diverse ecological conditions have likely contributed to regional differentiation in adaptive traits and breed characteristics. Although previous studies have identified candidate genes associated with cold adaptation in Toggenburg goats [12], systematic landscape genomic investigations of environmental adaptation in Chinese dairy goats across different regions remain limited. Meanwhile, horn type and coat color are also important phenotypic traits shaped during breed improvement. Chinese dairy goats have gradually developed a predominantly white coat color and differentiated into horned and polled types, with polled individuals generally being more compatible with the management requirements of modern intensive production systems [13]. Previous studies have demonstrated that genes such as *MC1R*, *ASIP*, *TYR*, and *MITF* play important roles in coat color determination in cattle [14], horses [15–17], sheep [18], and ducks [19], while *ZEB2*, *ERG*, and *TWIST1* are closely associated with horn types [20–22]. However, the genetic basis underlying horn type and coat color in Chinese dairy goats remains poorly understood, and their potential roles in environmental adaptation and breed differentiation have yet to be systematically characterized.

In this study, we analyzed whole-genome resequencing data from indigenous, cultivated, and introduced dairy goat populations to characterize the population structure and genetic diversity of Chinese dairy goats. We further applied machine learning approaches for Chinese dairy goat breed classification and informative SNP marker identification, and identified candidate genes and variants associated with environmental adaptation, horn type, and coat color. These findings provide an important scientific basis for precision breeding, breed identification, conservation, and sustainable utilization of Chinese dairy goat genetic resources.

## Materials and methods

### Sample collection and sequencing

In this study, blood or ear tissue samples were collected from six Chinese dairy goat breeds, including Laoshan (LS, *n* = 20), Wendeng (WD, *n* = 20), Ya'an (YA, *n* = 18), Hebei (HB, *n* = 20), Hongdong (HD, *n* = 30), and Henan (HN, *n* = 25), and sequenced on the DNBSEQ-T7 platform (BGI, Shenzhen, China). In addition, previously published and publicly available whole-genome datasets were incorporated into the analysis (Additional file 1: Table S1), including Australian Nubian (ANB, *n* = 5), Australian Alpine (AP, *n* = 7), Australian Saanen (AS, *n* = 8), Guanzhong (GZ, *n* = 6), Nubian (NB, *n* = 10), Toggenburg (TG, *n* = 6), Korean Saanen (HS, *n* = 10), New Zealand Saanen (XS, *n* = 6), Xinong Saanen (XN, *n* = 40), Yunshang Black (YSB, *n* = 6), Guishan (GS, *n* = 6), Longlin (LL, *n* = 15), and Bezoar (WL, *n* = 12).

### Variant detection and quality control

The quality of the raw sequencing reads was assessed using FastQC v0.12.1. Adapter removal and quality trimming were performed using fastp v0.24.0 [23] to obtain high-quality clean reads. The filtered reads were aligned to the Saanen reference genome (Saanen_v1, GCA_015443085.1) [24] using the Burrows–Wheeler Aligner (BWA) v0.7.17 [25]. Alignments were converted to BAM format and sorted using SAMtools v1.9 [26], and duplicate reads were marked using the MarkDuplicates module in the Genome Analysis Toolkit (GATK) v4.1.8 [27]. Single-nucleotide polymorphisms (SNPs) were identified using the HaplotypeCaller module in GATK v4.1.8, followed by hard filtering with the VariantFiltration module according to the GATK best practices [28] using the following criteria: *QD* < 2.0, *FS* > 60.0, *MQ* < 40.0, *MQRankSum* <−12.5, *ReadPosRankSum* < −8.0. Further quality control of SNPs was conducted using VCFtools v0.1.16 [29]. Missing genotypes were imputed using Beagle v5.4 [30] with a self-imputation strategy, followed by additional filtering in PLINK v1.9 [31] with the parameters (--*mind 0.1 --geno 0.1 --maf 0.05 --hwe 1e-6*) to retain high-quality genotypes. The retained high-quality SNPs were functionally annotated using ANNOVAR [32].

### Population structure and genetic relationship analysis

To investigate the genetic relationships and population structure among dairy goats, quality-filtered SNPs were used for population structure analyses. Pairwise genetic distances between individuals were calculated using PLINK v1.9, and a Neighbor-Joining (NJ) tree was constructed using MEGA v11 [33] and visualized using iTOL v7 (https://itol.embl.de). Principal component analysis (PCA) was performed using linkage disequilibrium (LD) pruned SNPs (--indep-pairwise 50 10 0.2) with PLINK v1.9 to calculate the first three principal components (PC1–PC3), which were visualized using R v4.3.3. Population genetic structure was inferred using ADMIXTURE v1.3.0 [34], with the number of ancestral clusters (K) ranging from 1 to 16. For each K value, five independent runs were performed with different random seeds, and the run with the lowest cross-validation (CV) error was selected as the result. The optimal K value was determined based on the minimum CV error across all runs, and ancestry proportions were visualized in R v4.3.3.

### Introgression and demographic history analysis

LD was evaluated by calculating the squared correlation coefficient (*r*^2^) between SNP pairs using PopLDdecay v3.40 [35] with default parameters. To infer the potential genetic introgression from introduced dairy goat breeds into Chinese dairy goats, migration events (*m* = 1–16) among 16 goat populations were modeled using TreeMix v1.13 [36], with Bezoar goats as the outgroup. The optimal number of migration edges was determined using the OptM R package based on the Δm statistic and the proportion of variance explained across a range of migration edges. Pairwise genetic differentiation among populations was estimated using VCFtools v0.1.16 with a sliding window size of 50 kb and a step size of 25 kb to calculate the fixation index (*F*_ST_) value. The qp3Pop program in ADMIXTOOLS [37] was used to compute the *f*₃-statistics for all population trios, with Bezoar goats as the outgroup. Historical effective population sizes (*Ne*) of European, African, Chinese dairy goats, and Bezoar goats were inferred using SMC++ v1.15.2 [38]. The mutation rate was set to 2.5 × 10⁻^8^ per generation, and the generation time was assumed to be two years [39]. The time scale for the demographic reconstruction ranged from 100 to 20,000 generations. Finally, runs of homozygosity (ROH) and genomic inbreeding coefficient based on runs of homozygosity (*F*_ROH_) were estimated using the detectRUNS package in R v4.3.3, with a minimum ROH length threshold of 0.1 Mb, to evaluate inbreeding levels across different populations and ROH length classes.

### Genetic diversity analysis

To investigate genetic diversity among different dairy goat populations, the proportion of polymorphic sites (*Pn*) was calculated for each population. The observed heterozygosity (*Ho*) and expected heterozygosity (*He*) of each population were estimated using PLINK v1.9, and the mean values were used to indicate the heterozygosity level of each population. Nucleotide diversity (π) was estimated using VCFtools v0.1.16 with a window size of 50 kb and a step size of 25 kb. ROHs were detected using PLINK v1.9, and *F*_ROH_ was defined as the proportion of the autosomal genome covered by ROHs for each individual.

### Machine learning analysis and SNP panel optimization

To evaluate the effects of machine learning models and SNP panel size on breed classification, 164 individuals from seven Chinese dairy goat breeds were analyzed. The genotype data were pruned using PLINK v1.9 (--indep-pairwise 50 10 0.2) based on linkage disequilibrium and converted into an allele dosage matrix (0/1/2 coding) for downstream analysis. The dataset was divided into training (80%) and testing (20%) sets by stratified sampling, and this process was repeated 10 times. SNP selection was performed only on the training set. Pairwise *F*_ST_ values were calculated using VCFtools v0.1.16, and SNPs were ranked according to the maximum *F*_ST_ value across all pairwise population comparisons. The top-ranked SNPs (*n* = 100, 200, 300, 500, 800, 1,000, 2000, 3,000, 5,000, 8,000, 10,000) were used to train five machine learning algorithms, including random forest (RF), k-nearest neighbors (KNN), extreme gradient boosting (XGBoost), decision tree (DT), and support vector machine (SVM). Model hyperparameter tuning was conducted using five-fold cross-validation within the training set, and performance was evaluated on the independent test set using accuracy, Cohen’s kappa coefficient (κ), and the area under the ROC curve (AUC) [40].

Based on comparative model performance, KNN was selected as the optimal classifier, and the top 800 SNPs were used as the initial feature set for subsequent feature selection and stability analysis. SNPs ranked by *F*_ST_ were used for stability selection, and those appearing in ≥ 70% of cross-validation repetitions were retained as the core SNP set. Permutation importance was then applied to rank the core SNPs, and nested subsets of increasing size were constructed to evaluate classification performance. The performance plateau was defined as the point at which incremental gains in accuracy, Cohen’s kappa (κ), and AUC were all < 0.005, and the corresponding subset was identified as the minimal SNP panel. The minimal SNP set was evaluated on an independent test set using confusion matrices and breed-level recall. Finally, the selected SNPs were annotated to genes using BEDTools v2.25.0 [41], and Kyoto Encyclopedia of Genes and Genomes (KEGG) pathway enrichment analysis of the candidate genes was performed using KOBAS [42].

### Environmental variables and gene–environment association analysis

To evaluate the impact of environmental factors on Chinese dairy goats, 19 bioclimatic variables (BIO1–BIO19) were obtained from WorldClim [43], covering the period from 1970 to 2000 at a spatial resolution of 5 arcminutes. Environmental values were extracted for each sampling location based on geographic coordinates. To reduce multicollinearity among variables, variance inflation factors (VIF) were calculated using the usdm package in R v4.3.3 [44], and variables with VIF < 10 were retained [45]. Gene–environment associations were assessed using the Latent Factor Mixed Model (LFMM) implemented in the lfmm package in R v4.3.3 [46], with the genotypes as response variables and the selected bioclimatic variables as explanatory variables. Population structure was accounted for by setting the number of latent factors to K = 5. SNPs significantly associated with at least one environmental variable were defined as environment-associated loci (EALs). Following previous LFMM studies, loci with |*Z*| ≥ 4 and Bonferroni-corrected *P* ≤ 0.05/*N*_pruned_ were retained as EALs [47], where *N*_pruned_ represents the number of independent SNPs obtained after LD pruning. To evaluate the joint effects of environmental variables on genetic variation, redundancy analysis (RDA) was performed using the LFMM-identified EALs as response variables and the selected bioclimatic variables as predictors [48]. A partial RDA was further conducted by conditioning on longitude and latitude to control spatial effects. Functional annotation of candidate regions was performed using BEDTools v2.25.0, and enrichment analyses were conducted using KOBAS.

### Candidate gene identification for horn type and coat color traits in dairy goats

Based on phenotypic data, dairy goats were classified into horned and polled groups, as well as white and non-white coat color groups. Selection signature analyses were performed using fixation index (*F*_ST_), nucleotide diversity estimator (θπ), and Z-transformed haplotype homozygosity (ZHp), and the top 5% outlier regions identified by each method were defined as candidate regions. Candidate regions were annotated using BEDTools v2.25.0, and functional enrichment of candidate genes was performed using KOBAS. To further refine candidate loci, overlapping genomic regions identified by multiple selection methods were assessed using *F*_ST_, θπ, ZHp, and Tajima’s D. Genome-wide association studies (GWAS) were performed using a mixed linear model implemented in GEMMA v0.98.3 [49], with principal component scores derived from PCA and the kinship matrix included as covariates. GWAS results were visualized using the CMplot package in R v4.3.3, and Bonferroni-corrected significance thresholds (0.01/*n* and 0.05/*n*) were applied, with a suggestive threshold (1/*n*) used to identify potentially associated loci. Genes located within 100 kb upstream and downstream of significant SNPs were annotated as candidate genes. LD block structures of significant loci were visualized using LDBlockShow v1.40 [50] to characterize the genetic architecture underlying horn type and coat color.

### Functional prediction of candidate variants for horn type

Based on the above analyses, *ERG* was identified as a candidate gene associated with horn type. The identified missense variants were further validated in expanded horned and polled goat populations using the GoatVar database (http://animal.omics.pro/code/index.php/goatvar). Primers for polymerase chain reaction (PCR) amplification were designed using the NCBI Primer-BLAST online tool: forward primer (F) 5′‑GTAAGTGCCCAGATGCGAAG‑3′ and reverse primer (R) 5′‑GTTCCGTTTCCTTTCCCCAC‑3′. PCR amplification followed by Sanger sequencing was performed to validate the variants. ERG protein sequences from 25 species were retrieved from the UniProt database (https://www.uniprot.org/uniprot). Multiple sequence alignment was performed using MEGA v11, and sequence conservation was visualized using WebLogo (https://weblogo.berkeley.edu/logo.cgi). To evaluate the potential structural effects of missense variants, protein structures before and after amino acid substitutions were modeled using ChimeraX [51]. The functional impact of amino acid substitutions was further predicted using PROVEAN [52].

## Result

### Genomic variation characteristics and data quality

A total of 12,699.5 Gb of sequencing data were generated from 270 goats, with an average sequencing depth of 15.83 × (5.47 × –38.64 ×). The reads were aligned to the Saanen_v1 reference genome (GCA_015443085.1), with an average mapping rate of 99.64% and a genome coverage of 98%. After variant quality control, a total of 12,283,957 high-quality SNPs were identified. Functional annotation showed that more than 91% of SNPs were located in non-coding regions, including 54.73% intergenic and 36.92% intronic variants. Only 0.66% were located in exonic regions, comprising 27,413 nonsynonymous and 53,320 synonymous mutations. The average transition/transversion (Ts/Tv) ratio was 2.47 (Additional file 1: Table S2).

### Population genetic structure analysis

**Fig. 1:**
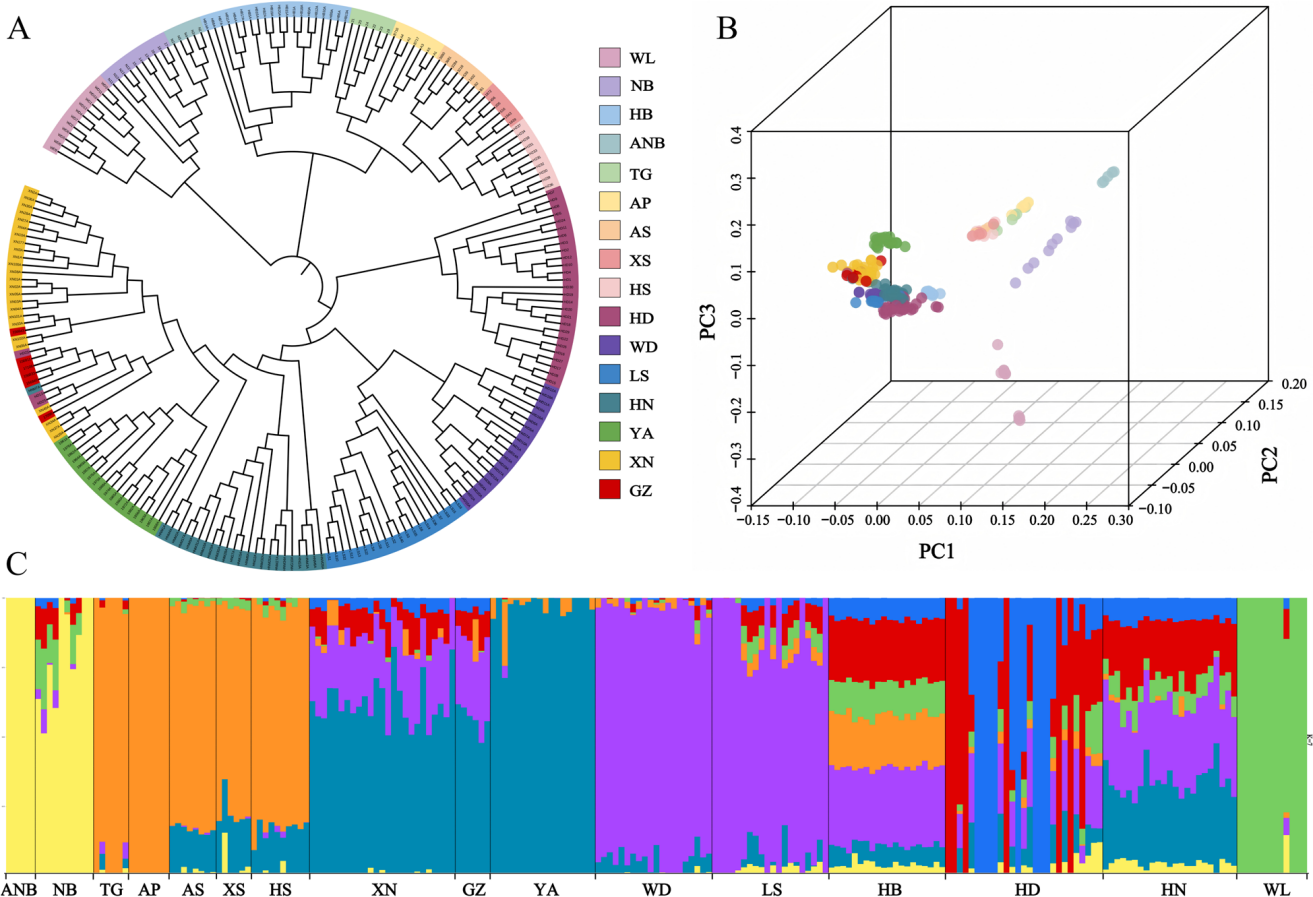
Population genetic structure of goat breeds. **A** Neighbor-joining (NJ) phylogenetic tree, with colors representing different populations. **B** Three-dimensional principal component analysis (PCA) based on PC1–PC3. **C** Population structure inferred by ADMIXTURE at the optimal K = 7

The NJ tree revealed that the 16 goat populations clustered into three major branches (Fig. 1A). Bezoar and Nubian goats formed the first branch; European (Toggenburg, Alpine, and Saanen) together with Hebei dairy goats clustered in the second branch; and Chinese dairy goat breeds constituted the third branch. Within the Chinese dairy goat branch, Wendeng and Laoshan formed one sub-branch, Hongdong clustered independently, and Henan, Ya'an, Guanzhong, and Xinong Saanen formed another sub-branch. Consistent with the NJ tree, PCA separated the 16 populations into three clusters corresponding to Bezoar and Nubian, European, and Chinese dairy goats (Fig. 1B). PCA revealed clustering consistent with the geographic origins of Chinese dairy goats (Additional file 2: Fig. S1).

To explore the population structure of dairy goats in China, ancestral components were inferred for K = 1–16. The optimal number of clusters (K = 7) was determined based on the lowest cross-validation error (Additional file 1: Table S3). At K = 7, African (NB, ANB), European (TG, AP, AS, HS, XS), Chinese (XN, GZ, YA, WD, LS, HB, HD, HN), and Bezoar goats (WL) showed genetic separation into distinct ancestral components (Fig. 1C). Most Chinese dairy goat breeds carried Saanen components, while Hebei dairy goats also carried a minor European ancestry component. Xinong Saanen and Guanzhong dairy goats showed highly similar ancestry profiles, while Wendeng and Laoshan dairy goats showed comparable genetic composition. Ya'an dairy goats exhibited a relatively homogeneous genetic structure. These results indicated genetic differentiation among Chinese dairy goats from different geographical regions.

### Introgression and demographic history analysis

**Fig. 2:**
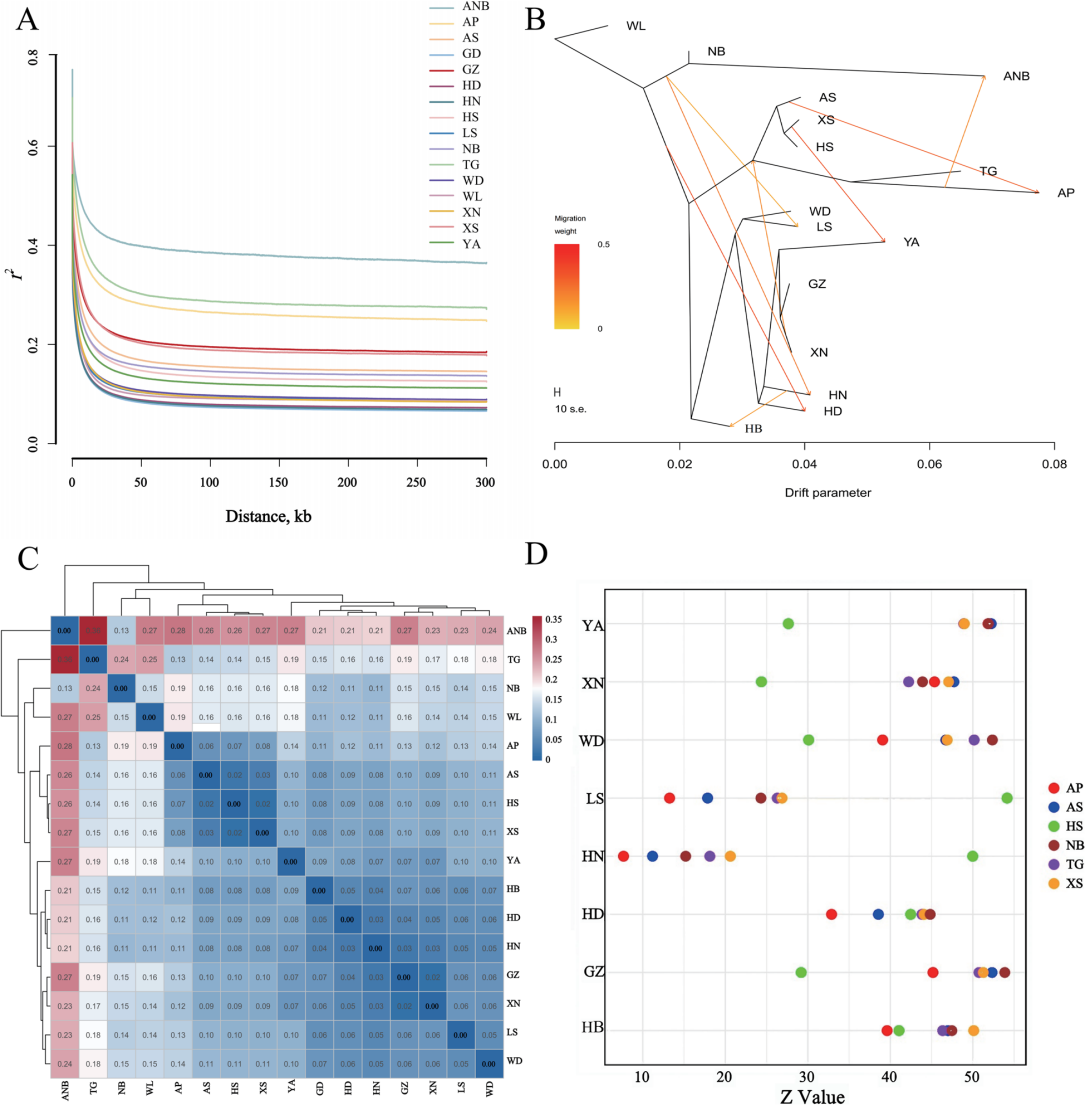
Introgression and demographic history analyses of dairy goat populations. **A** Linkage disequilibrium (LD) decay in different populations. **B** TreeMix-inferred gene flow among populations, with arrows indicating inferred migration events among populations. **C** Pairwise genetic differentiation (*F*_ST_) among populations. **D** Outgroup *f*₃-statistics between introduced dairy goat breeds and Chinese dairy goat populations

Linkage disequilibrium (LD) decayed rapidly within 0–50 kb in all populations and reached a plateau at approximately 200 kb (Fig. 2A). European (TG, AP, AS, HS, XS) and African (NB, ANB) dairy goats showed relatively slower decay, whereas Chinese dairy goat populations, particularly HB, HN, and HD, exhibited faster decay. The TreeMix maximum-likelihood analysis with Bezoar goats as the outgroup revealed a hierarchical genetic structure among African, European, and Chinese dairy goats. Based on the selected migration model (*m* = 8) (Additional file 2: Fig. S3), several gene flow events were inferred, including migration edges from Nubian to Laoshan (LS), Hongdong (HD), and Hebei (HB); from Saanen to Alpine (AP) and Ya'an (YA); and from Henan (HN) to Hebei (HB) (Fig. 2B). The outgroup-*f*_3_ statistics indicated significant introgression signals (*Z* > 3) among Saanen, Alpine, Toggenburg, Nubian, and Chinese dairy goats, suggesting historical or recent gene flow across populations (Fig. 2D). Consistently, European and Nubian goats exhibited higher genetic differentiation from Chinese dairy goats, whereas differentiation among Chinese dairy goat breeds was relatively low (Fig. 2C). Historical demographic analyses revealed a pronounced historical bottleneck in Chinese dairy goats, with consistently lower effective population sizes (*Ne*) compared with European and African dairy goats (Additional file 2: Fig. S5). In contrast, the high proportion of short ROH segments and lower overall inbreeding coefficients indicate relatively high recent genetic diversity and substantial recent gene flow within Chinese dairy goat populations (Additional file 2: Fig. S6).

### Genetic diversity among dairy goat populations

Genetic diversity analysis showed that Chinese dairy goats exhibited higher polymorphism and heterozygosity than African (NB, ANB) and European (AP, TG, HS, AS, XS) dairy goats (Table 1), with indigenous Chinese dairy breeds generally showing greater genetic diversity than cultivated populations, particularly Hongdong (HD) and Henan (HN). Nucleotide diversity (π) was lower in European (0.00130) and African (0.00111) goats than in Chinese dairy goats (0.00153), with Hongdong (0.00158) and Henan (0.00159) exhibiting the highest diversity among all populations. In addition, *F*_ROH_ values were higher in European and Nubian populations compared with Chinese dairy goats (Additional file 2: Fig. S8). Among Chinese dairy breeds, Laoshan (0.065), Hebei (0.084), Wendeng (0.089), and Henan (0.096) showed relatively low inbreeding levels, whereas Ya'an (0.146) and Guanzhong (0.147) exhibited higher inbreeding levels.

**Table 1:** Average genetic diversity indices for each population

Breed	*Pn*^1^	*Ho*^2^	*He*^3^	*F*_ROH_^4^	π^5^
ANB	0.405	0.164	0.146	0.272	0.00089
NB	0.727	0.242	0.230	0.115	0.00132
AP	0.565	0.196	0.188	0.263	0.00110
TG	0.573	0.241	0.191	0.188	0.00114
HS	0.779	0.259	0.248	0.106	0.00142
AS	0.749	0.255	0.243	0.110	0.00141
XS	0.712	0.264	0.240	0.105	0.00143
LS	0.915	0.282	0.273	0.065	0.00151
WD	0.908	0.277	0.270	0.089	0.00149
YA	0.850	0.262	0.257	0.146	0.00143
GZ	0.787	0.279	0.262	0.147	0.00156
XN	0.965	0.287	0.282	0.118	0.00155
HD	0.997	0.282	0.289	0.106	0.00158
HN	0.978	0.280	0.289	0.096	0.00159
HB	0.944	0.267	0.278	0.084	0.00154
WL	0.728	0.178	0.225	0.187	0.00128

^1^The proportion of polymorphic markers

^2^Observed heterozygosity

^3^Expected heterozygosity

^4^The average inbreeding coefficient

^5^Nucleotide diversity

### Machine learning analysis and SNP panel optimization

**Fig. 3:**
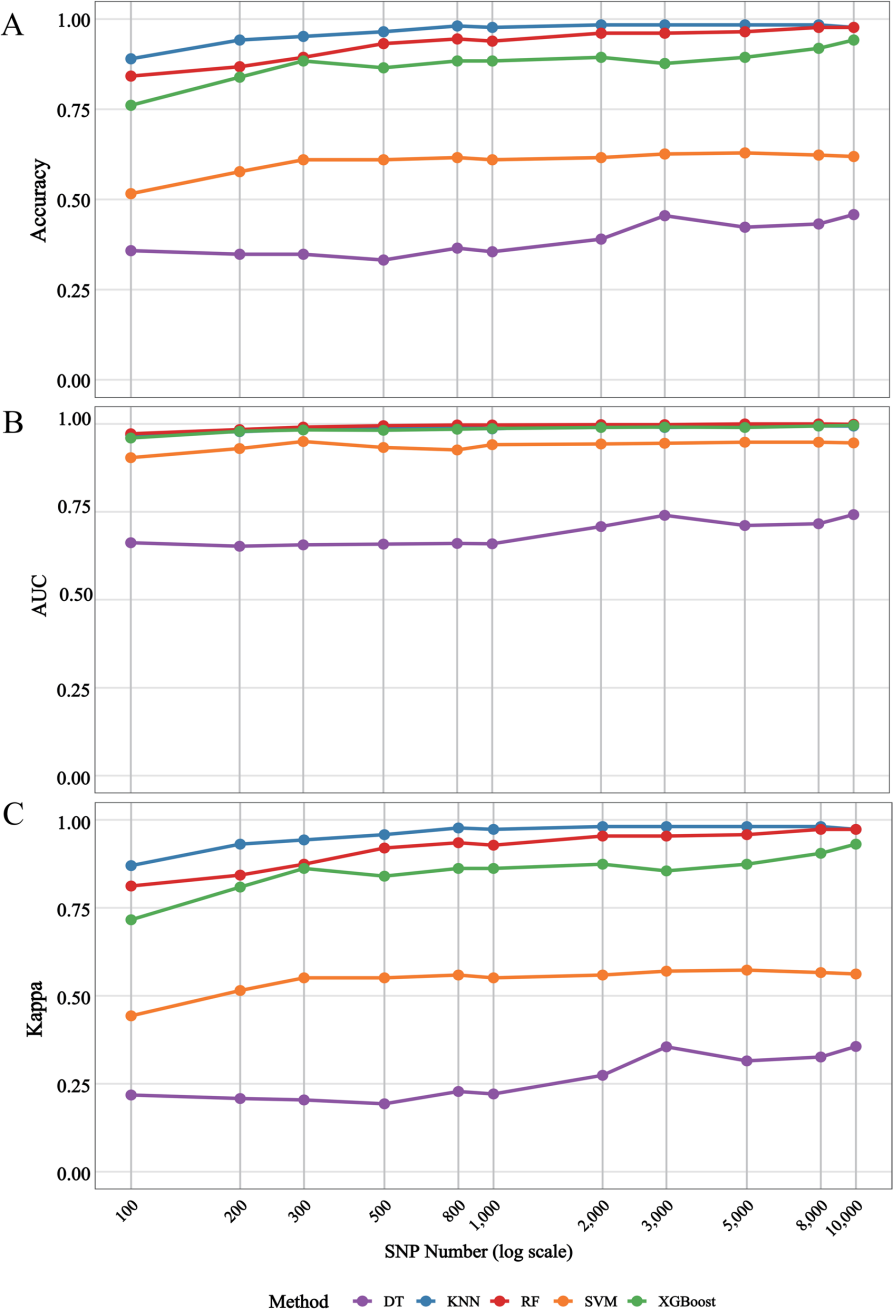
Performance comparison of five machine learning algorithms based on different SNP sets. **A** Accuracy. **B** Area under the curve (AUC). **C** Cohen’s kappa coefficient (κ)

Five machine learning algorithms were evaluated for Chinese dairy goat breed classification across SNP panels of increasing size (Additional file 1: Table S6). Overall, classification accuracy improved as the number of SNPs increased (Fig. 3A–C). KNN and RF consistently outperformed the other models, whereas SVM and DT showed relatively lower performance. Notably, KNN achieved 98.1% ± 1.7% accuracy using 800 SNPs, close to its maximum performance (98.4% ± 3.1%) observed with larger SNP panels. RF showed strong performance, reaching 97.7% ± 3.4% accuracy with 8,000 SNPs, while XGBoost exceeded 94% accuracy only at 10,000 SNPs. In general, KNN showed the best performance for Chinese dairy goat breed classification, particularly when the number of informative SNPs is limited (Additional file 2: Fig. S9).

**Fig. 4:**
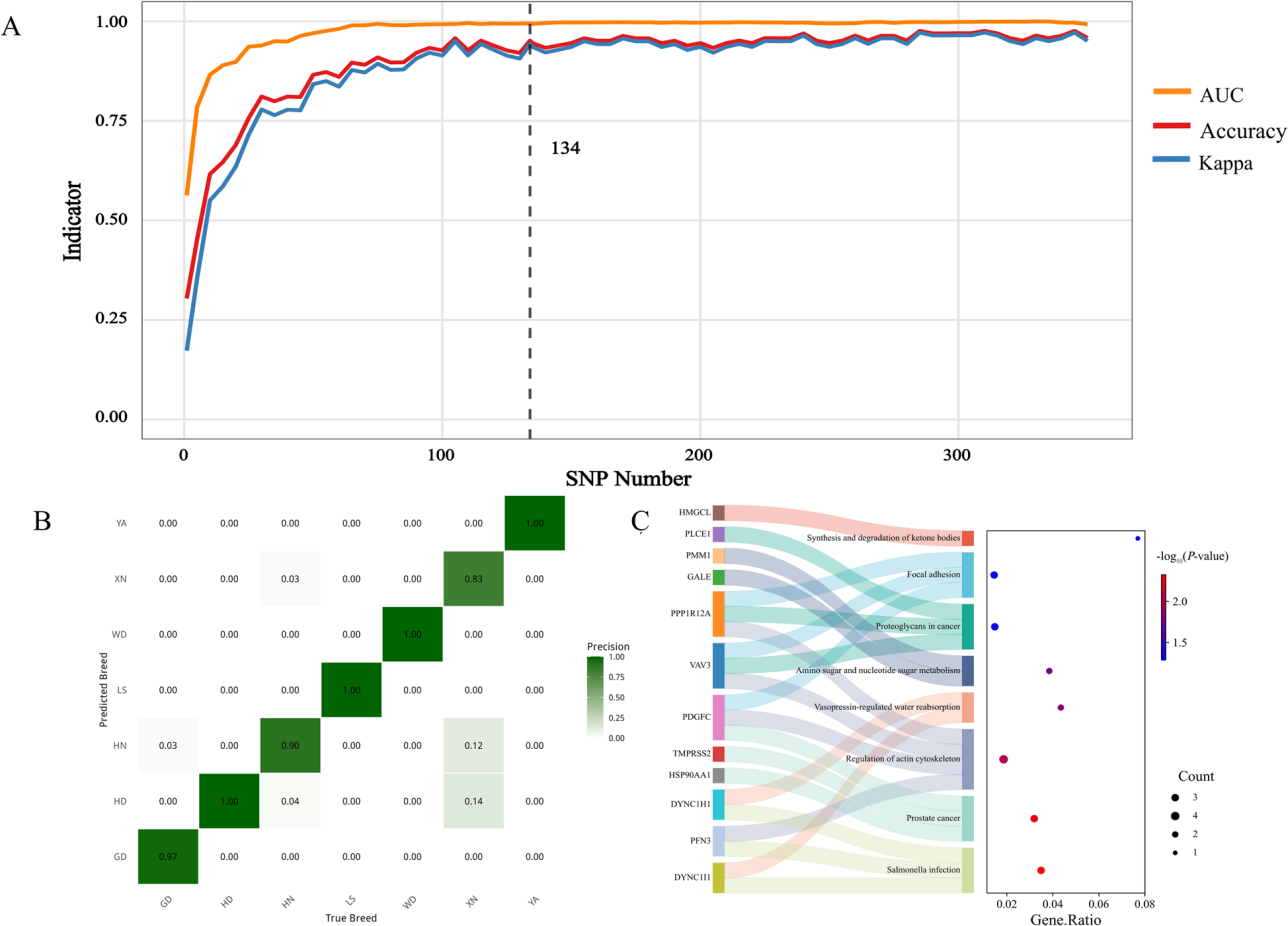
Molecular marker identification of Chinese dairy goat breeds. **A** Performance metrics of the KNN for different SNP sets, with red, orange, and blue representing accuracy, area under the curve (AUC), and Cohen’s kappa coefficient (κ), respectively. **B** Classification confusion matrix. **C** KEGG enrichment analysis of 134 SNPs annotated genes

To construct a cost-effective breed classification panel, KNN was selected as the optimal classifier for subsequent optimization analyses. The top 800 SNPs ranked by *F*_ST_ were subjected to stability selection, resulting in 351 core SNPs that appeared in ≥ 70% of replicates. This core set retained strong predictive performance on the independent test set (accuracy = 0.945, AUC = 0.997), comparable to that of the top 800 SNP set (Additional file 2: Fig. S10). Subsequently, permutation importance ranking was applied to construct SNP subsets of varying sizes, and a minimal subset of 134 SNPs achieved robust classification performance (Fig. 4A, accuracy = 0.951, AUC = 0.995), comparable to that of the 351-core SNP set (Additional file 1: Table S7). Validation on the independent test set confirmed that the 134-SNP panel performed similarly to both the 351-core and top 800 SNP sets. Confusion matrix analyses showed that most breeds were correctly classified (Fig. 4B, Additional file 2: Figs. S11 and S12). These results suggest that the minimal SNP panel reduced the number of markers while still maintaining high classification accuracy.

Functional annotation of the 134 SNPs identified 149 candidate genes (Additional file 1: Tables S8–S9). KEGG enrichment analysis indicated that these genes were significantly enriched in pathways related to cell signal transduction, cytoskeleton remodeling, and immune processes, such as Rap1 signaling pathway, Salmonella infection, Prostate cancer, and Regulation of actin cytoskeleton (Fig. 4C). These results indicate that the selected SNPs not only enable effective breed classification, but may also be associated with physiological regulation and adaptive traits in Chinese dairy goats.

### Landscape genome and environmental association analysis

**Fig. 5:**
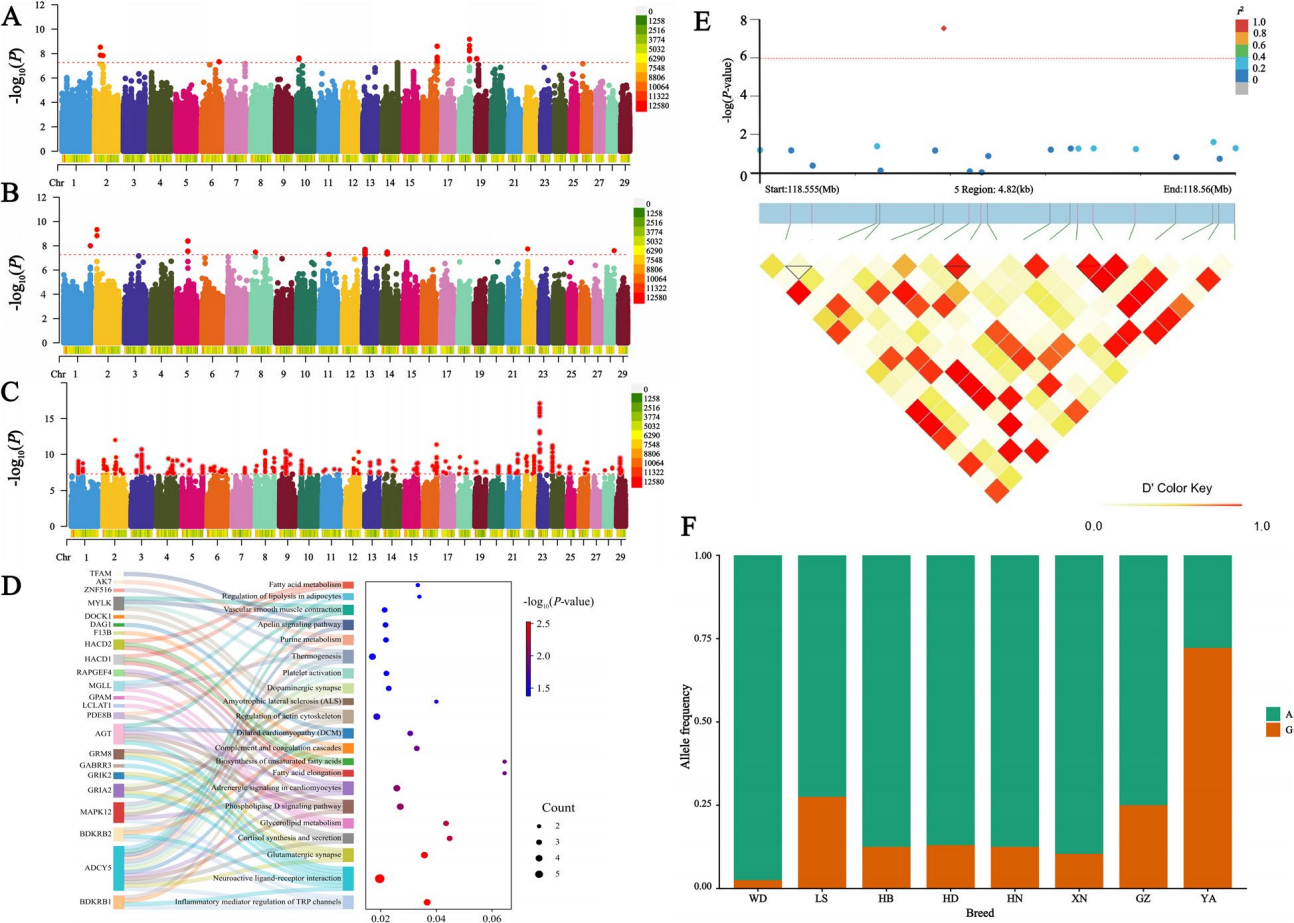
Environmental association analysis. **A**–**C** LFMM association analyses of BIO3, BIO8, and BIO9, respectively. **D** KEGG enrichment analysis of the candidate genes. **E** Linkage disequilibrium (LD) pattern of the *HDAC10* gene region. **F** Allele frequency distribution of chr5:118557145 A > G across different Chinese dairy goat population

To investigate the adaptive genetic mechanisms of Chinese dairy goats under diverse climatic conditions, genomic and environmental data were integrated for landscape genomic analysis. Environmental variables were extracted from eight geographic sampling locations, and correlation analysis revealed strong collinearity among bioclimatic variables (Additional file 2: Fig. S13). After variance inflation factor filtering (VIF < 10), three relatively independent variables, Bio3 (Isothermality), Bio8 (Mean Temperature of Wettest Quarter), and Bio9 (Mean Temperature of Driest Quarter), were retained for subsequent analyses (Additional file 2: Figs. S14 and S15). Genome–environment association analysis using LFMM identified 654 significantly associated SNPs corresponding to 167 candidate genes for Bio3, Bio8, and Bio9 (Fig. 5A–C; Additional file 1: Tables S10 and S11). KEGG enrichment analysis revealed significantly enriched pathways related to inflammatory mediator regulation of TRP channels, glutamatergic synapse, vascular smooth muscle contraction, adrenergic signaling in cardiomyocytes, fatty acid elongation, thermogenesis, and fatty acid metabolism (Fig. 5D). Among the candidate genes, *ADCY5*, *GPAM*, *MGLL*, *HACD1*, and *HACD2* were involved in energy metabolism; *AGT*, *BDKRB1*, and *BDKRB2* were associated with physiological homeostasis under heat and drought stress; and *GRIA2*, *GRM8*, *IL1RL1*, and *TRIL* were involved in nervous system and immune regulation. In addition, a *HOXD* gene cluster (*HOXD8*–*HOXD13*) associated with trunk and limb morphogenesis during embryonic development was identified among the candidate genes.

Notably, a significant missense mutation (chr5:118557145 A > G) was identified in the coding region of *HDAC10*, resulting in a substitution of glutamine (Q) by arginine (R) at position 123 (Q123R) (Fig. 5E). The allele frequency of this variant showed a clear geographic pattern. The G allele reached 0.72 in Ya'an dairy goats, whereas it was only about 0.11 in HB, HD, HN, and other northern Chinese populations (Fig. 5F). Partial redundancy analysis (RDA) based on environmentally associated SNPs identified by LFMM revealed a highly significant model (*P* = 0.001). The first RDA axis (RDA1) explained 11.33% of the genetic variation and clearly separated Ya'an dairy goats from the other populations (Additional file 2: Fig. S16), suggesting that temperature related environmental factors have played an important role in shaping the adaptive genetic structure of Chinese dairy goats.

### Candidate gene analysis of horn type in dairy goats

**Fig. 6:**
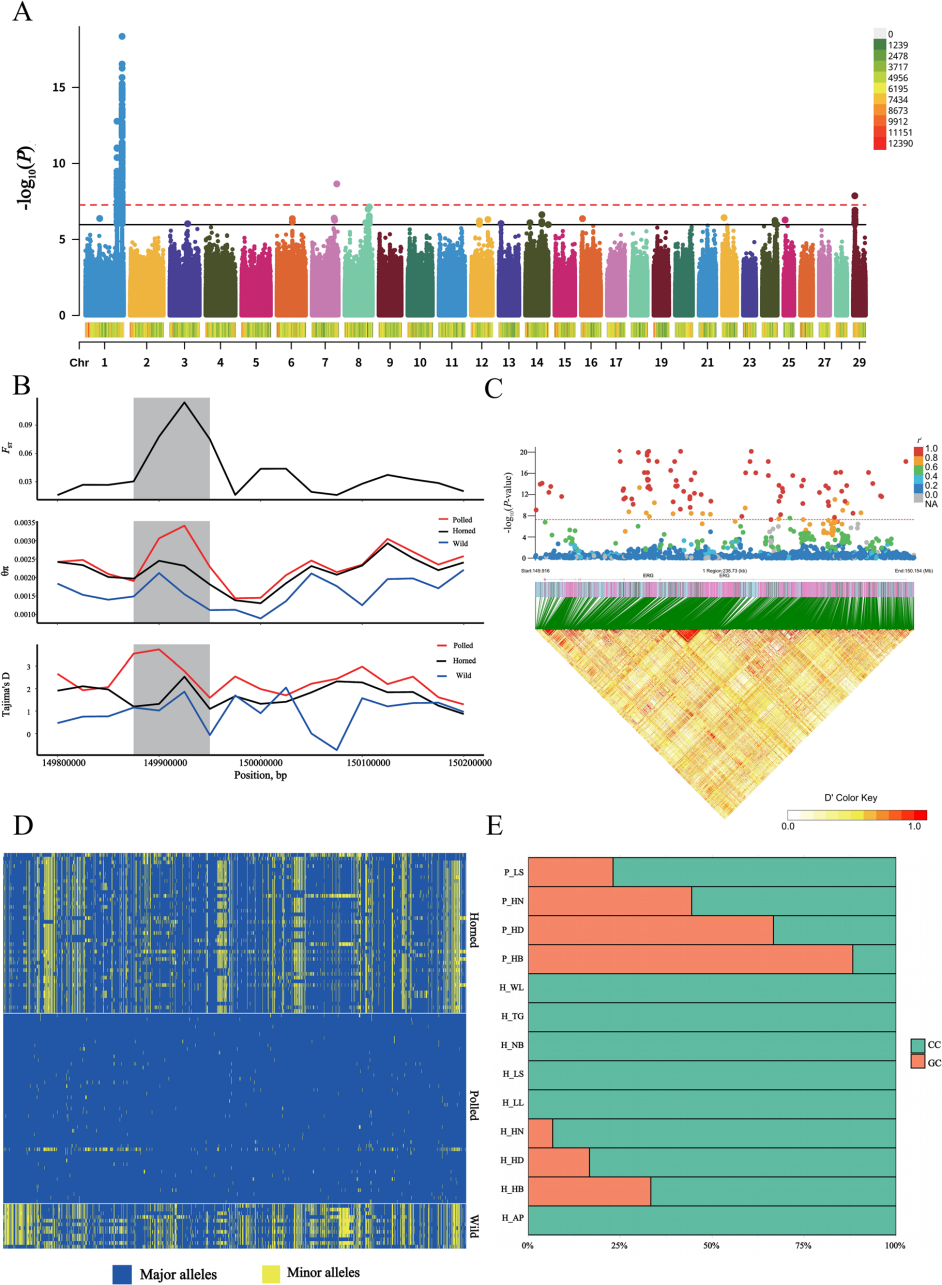
Genetic analysis of horn type in goats. **A** Genome-wide association study (GWAS) of horn type. **B** Selection signal analysis in the chr1:149.8–150.2 Mb region. **C** Linkage disequilibrium (LD) pattern in the *ERG* gene region. **D** Haplotype heatmap of the *ERG* gene, with blue and yellow indicating major and minor haplotypes in the polled population, respectively. **E** Genotype frequencies of the missense mutation chr1:149921788 C > G across different breeds

Selection signal analyses between horned and polled dairy goats were performed using *F*_ST_, θπ, and ZHp (Additional file 1: Tables S12–S14, Additional file 2: Fig. S17). The top 5% overlapping regions identified by all three methods were considered candidate regions, which were further annotated to identify a total of 608 candidate genes (Additional file 1: Table S15). Functional enrichment analysis revealed 28 significant KEGG pathways (Additional file 2: Fig. S18), including JAK-STAT, mTOR, pluripotency regulation, neurotrophin, HIF-1, and Toll-like receptor signaling pathways. Several genes previously associated with horn phenotype were identified, including *FOXL2* and *RXFP2*, with mutations in *RXFP2* known to cause the polled phenotype. In addition, other candidate genes involved in horn development included *MRPS22*, *ERG*, *KCNJ15*, *FOXO1*, *ALX1*, *SHH*, and *CHD7*. GWAS of horn type identified 284 significant SNPs, which were annotated to 53 genes (Fig. 6A, Additional file 1: Tables S16 and S17). Two major association peaks were detected on chr1 at 128.87–129.46 Mb and 149.79–150.14 Mb (Fig. 6C, Additional file 2: Fig. S20), which contained candidate genes including *RBP2*, *COPB2*, *MRPS22*, *KCNJ15*, and *ERG*.

Notably, *ERG* showed a strong selection signal, and compared with horned goats, the polled population exhibited higher θπ and Tajima’s D in this region (Fig. 6B). The haplotype analysis revealed conserved haplotypes in the *ERG* region among horned goats, with high linkage disequilibrium (Fig. 6C and D). Interestingly, a missense mutation (chr1:149921788 C > G) within *ERG* was identified among the significantly associated SNPs and showed clear differentiation between horned and polled goats. The genotype frequency analysis showed that most horned individuals carried the CC genotype, whereas polled goats were predominantly GC heterozygotes (Fig. 6E). The C allele frequency was 97.3% in horned goats, whereas the G allele frequency was 2.7%. In contrast, the C and G allele frequencies in polled goats were 70.6% and 29.4%, respectively (Additional file 2: Fig. S21). Moreover, this locus also showed a homozygous CC genotype in other horned breeds, including Longlin, Leizhou, Cashmere, and Tibetan goats (Additional file 2: Fig. S22), suggesting that the G allele may be associated with the polled phenotype.

**Fig. 7:**
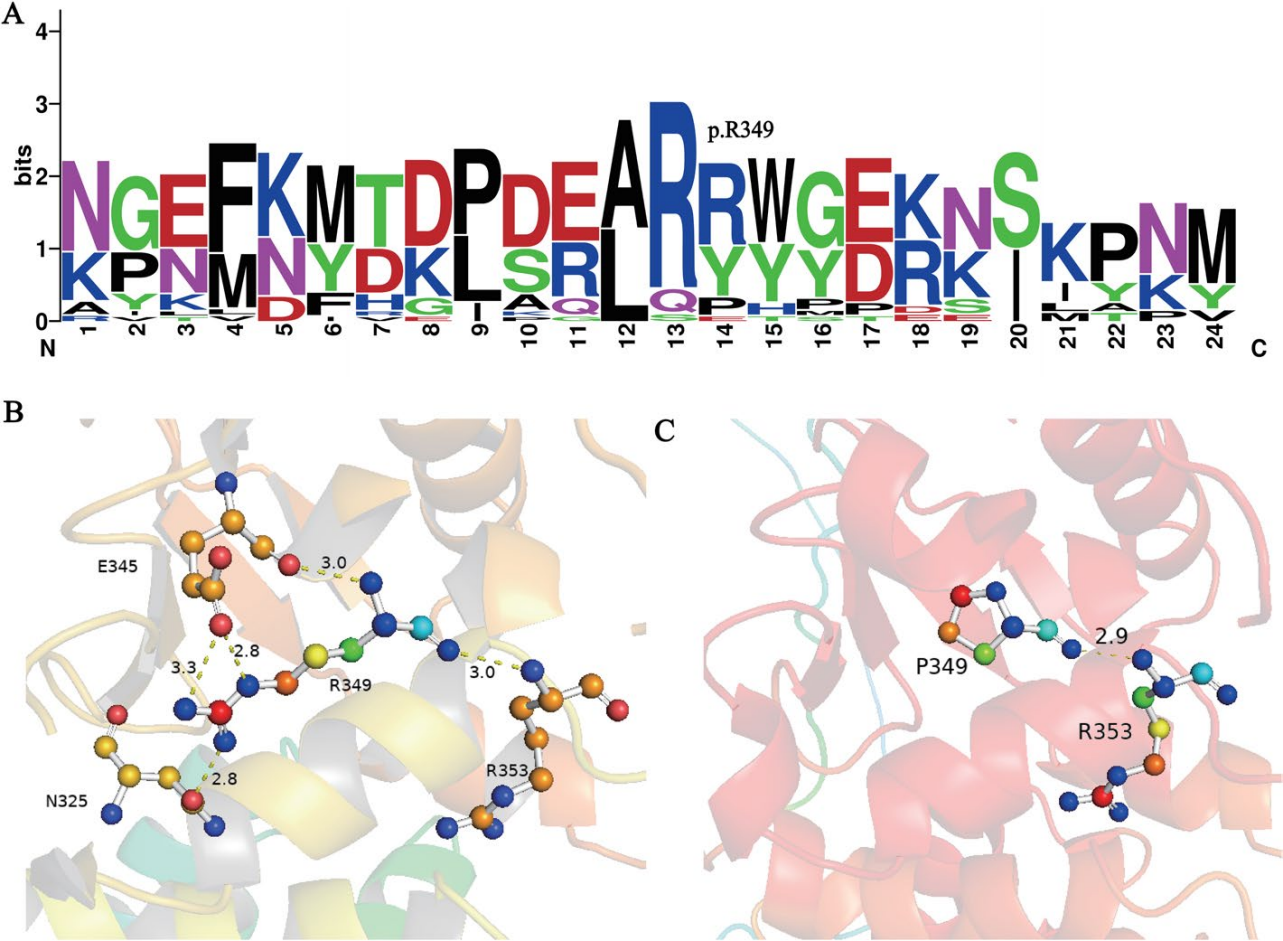
Structural analysis of ERG protein mutation sites. **A** Amino acid sequence conservation of ERG across different species. **B** Predicted secondary structure of the wild-type ERG protein, with yellow dotted lines indicating hydrogen bonds. **C** Predicted secondary structure of the mutant ERG protein with the arginine to proline substitution, with yellow dotted lines indicating hydrogen bonds

Moreover, the C > G variant at chr1:149921788 results in an arginine to proline substitution at amino acid position 349 (p.R349P). Comparative sequence analysis across 25 species indicates that this locus is highly conserved among cattle, goat, and sheep (Fig. 7A, Additional file 2: Fig. S23). The potential impact of the p.R349P missense mutation on ERG protein function was further evaluated in horned and polled populations. Structural analysis indicated that the arginine to proline substitution disrupted hydrogen bonds and salt-bridge interactions and introduced steric clashes at this position (Fig. 7B and C). PROVEAN predicted that the R349P is a deleterious mutation (score = −6.861; threshold < −2.5), suggesting a potential functional impact on horn phenotype differentiation.

### Analysis of candidate genes associated with coat color in dairy goats

**Fig. 8:**
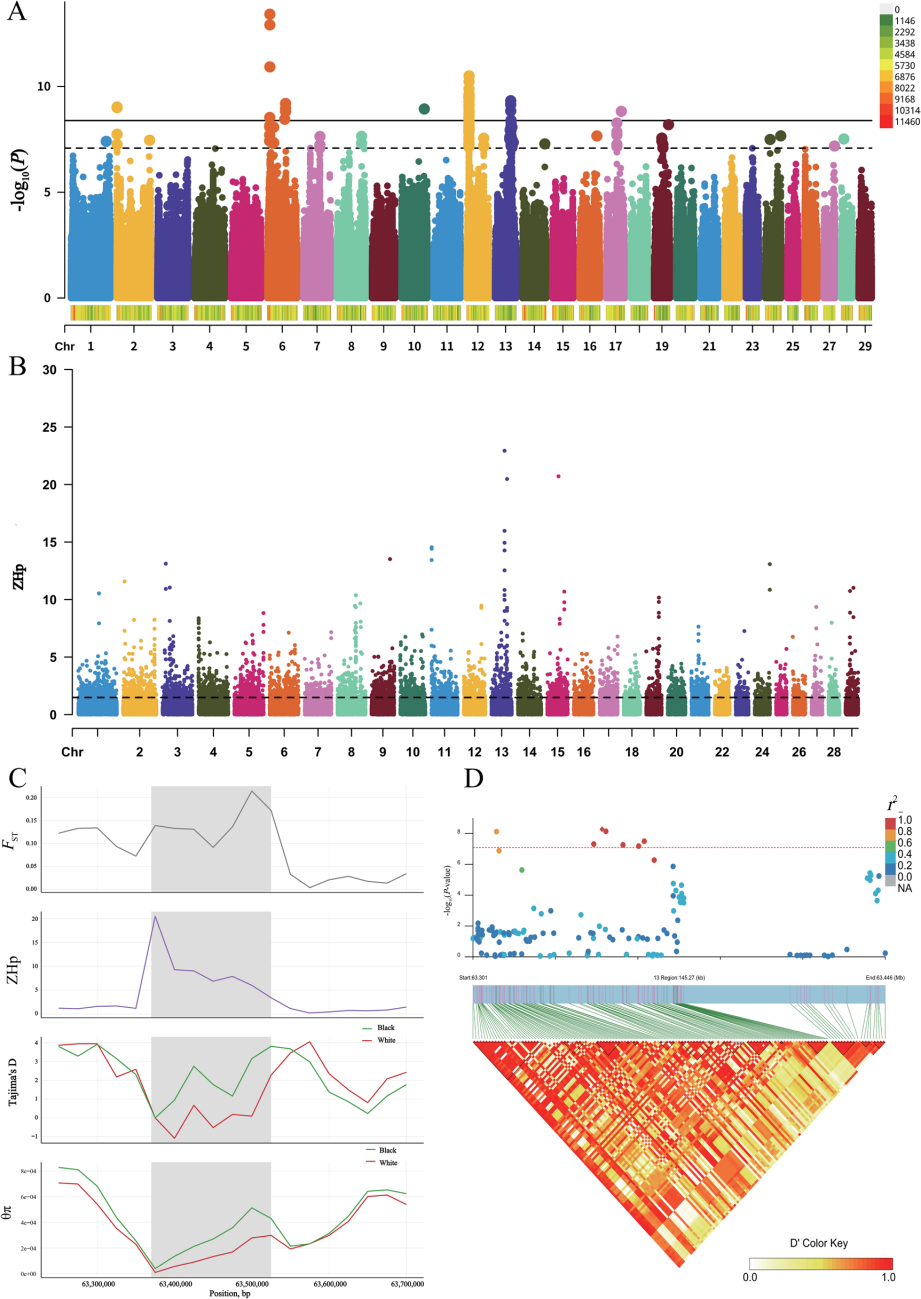
Analysis of coat color traits in goats. **A** Genome-wide association study (GWAS) of coat color. **B** ZHp-based selection signal analysis. **C** Comparison of four selection signal detection methods in the 63.3–63.7 Mb region on chr13. **D** Linkage disequilibrium (LD) block analysis for the 63.3–63.7 Mb region on chr13

Chinese dairy goats have gradually developed a predominant white coat color phenotype under long-term artificial selection. In this study, selection signature analyses combined with GWAS were performed to identify candidate genes associated with coat color (Fig. 8A and B, Additional file 2: Figs. S24 and S25). Selection signature analyses identified 76 genes consistently detected by all three methods, while genes identified by at least two methods were retained for downstream analyses (Additional file 1: Tables S18–S21). GWAS identified 468 significant SNPs that were annotated to 82 genes (Additional file 1: Tables S23–S24). KEGG pathway enrichment analysis of candidate genes revealed significant enrichments in melanogenesis, Hedgehog signaling, TGF-β signaling, cellular senescence, and cell cycle pathways (Additional file 1: Table S22), involving genes such as *ASIP*, *AHCY*, *ITCH*, *KIT*, *MITF*, *TYR*, *PMEL*, *E2F2*, *MYBL2*, and *TYRP1*. A strongly selected and highly differentiated region was identified on chr13 (63.35–63.55 Mb), with clear genetic divergence between black and white coated groups (Fig. 8C). GWAS detected a significant association peak overlapping this region, which harbors *ASIP* and *AHCY* (Fig. 8D).

**Fig. 9:**
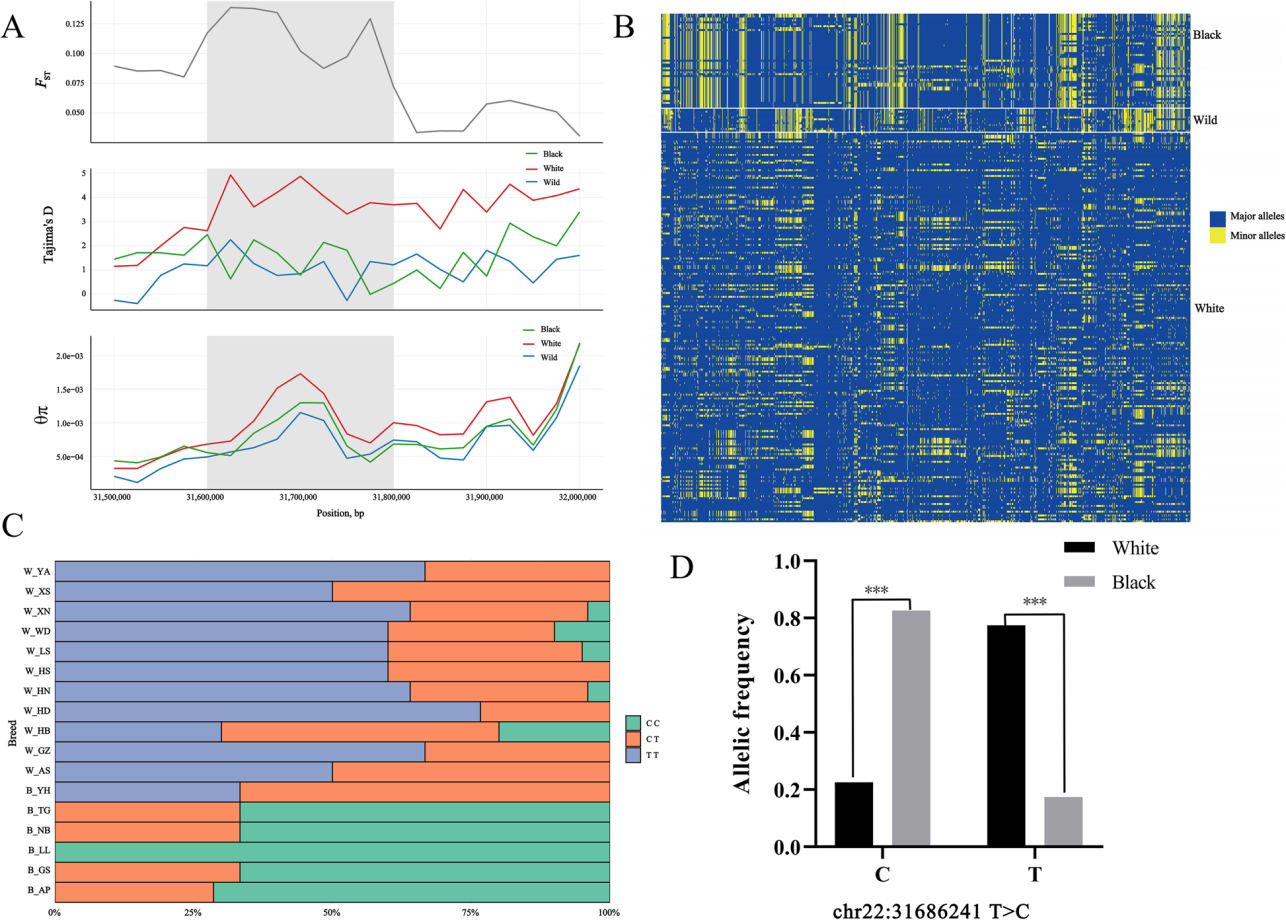
Fine mapping of the *MITF* gene. **A** Selection signal analysis in the 31.5–32 Mb region on chr22. **B** Haplotype heatmap of the *MITF* gene, where blue and yellow represent the major and minor alleles in the coat color groups, respectively. **C** Genotype frequencies of the missense mutation chr22:31686241 T > C among different goat breeds. **D** Allele frequency comparison of C and T alleles between white and black coat color groups (^***^P < 0.001)

Additionally, a strong selection signal was detected on chr22 (31.6–31.8 Mb) within the *MITF* gene region. In this region, the white coat population exhibited higher θπ and Tajima’s D than the black and other color populations (Fig. 9A), together with clear genetic differentiation between the two groups (Fig. 9B). A missense mutation (chr22:31686241 T > C) was identified in *MITF*, resulting in an isoleucine to valine substitution at position 13 (I13V). Genotype frequency analysis showed that TT and CT genotypes were predominantly observed in the white coat population (Fig. 9C and D). This variant may be associated with phenotypic differentiation between white and black coat colors.

## Discussion

Dairy goats are widely used for milk production and are adapted to a wide range of production systems. More than 60 dairy goat breeds are currently distributed worldwide, with approximately 216 million animals across 115 countries and regions (FAO, 2024). China introduced European dairy goat breeds in the early twentieth century and crossbred them with indigenous goats, resulting in improved production performance and the formation of regionally differentiated dairy goat populations. However, insufficient conservation and utilization of these genetic resources have reduced the scale of some breeds and increased genetic admixture, posing challenges for germplasm conservation and precision breeding. In this study, we used whole-genome resequencing to characterize the genetic structure and diversity of Chinese dairy goats and identify breed-specific molecular markers, providing genomic resources for germplasm conservation, molecular breeding, and future genetic improvement.

### Population genetic differentiation and evolutionary history of Chinese dairy goats

The genetic structure of Chinese dairy goats may be influenced by historical introgression, regional breeding practices, and long-term artificial selection. Population genetic analyses revealed that most Chinese dairy goat breeds carry Saanen ancestry, with a minor European ancestry component also detected in Hebei dairy goats. Both outgroup-*f*₃ statistics and migration analyses further indicated multiple historical gene flow events from European and African dairy goats into Chinese populations. These findings are consistent with historical records of crossbreeding between introduced dairy breeds and indigenous goats, providing molecular evidence for the formation of Chinese dairy goat genetic backgrounds. Despite this complex admixture history, the clustering patterns in the NJ tree and PCA still show clear geographic structure, suggesting that geographic separation, regional breeding, and local adaptation have contributed to population differentiation. However, the relatively low *F*_ST_ values indicate that historical exchange of breeding stock across production regions may have facilitated gene flow among some populations, which is consistent with previous studies [6]. Moreover, the relatively high levels of inbreeding detected in some breeds may reflect restricted population size or prolonged artificial selection, consistent with the historical breeding status of Chinese dairy goats.

Interestingly, although Chinese dairy goats generally exhibited lower *Ne* than European and African dairy goats, they still showed relatively high levels of contemporary genetic diversity. This pattern may be associated with recent gene flow among African, European, and Chinese dairy goat lineages during modern breeding. In addition, gene flow within Chinese dairy goat populations and the predominance of short ROH segments suggest that historical recombination and complex demographic processes may have contributed to maintaining allelic diversity [53]. Future studies incorporating a broader range of dairy goat breeds will further clarify their evolutionary history. Overall, these findings explain the relatively high genomic diversity in Chinese dairy goats and provide a genetic basis for future improvement and environmental adaptation.

### Comparison of machine learning models and SNP panel optimization

Breed identification is important for the conservation of local breeds, the evaluation of genetic resources, and breeding programs. However, Chinese dairy goat breeds exhibit low genetic differentiation and lack distinct phenotypic traits, making phenotype-based classification unreliable. SNP markers offer an effective method for breed identification. As a widely used measure of population genetic differentiation, *F*_ST_ has been applied to identify informative SNPs and characterize population structure [54, 55]. This study selected SNP features based on *F*_ST_ and compared the classification performance of five machine learning algorithms. KNN and RF consistently achieved high performance across different SNP panel sizes, while XGBoost also performed well with larger SNP sets, indicating better adaptability to high-dimensional genomic data and nonlinear relationships among loci. In contrast, SVM and DT showed lower performance, which may be related to limited sample size and reduced robustness under complex data structures. These results are consistent with previous studies showing that KNN performs well under low marker densities [56], suggesting its advantages in constructing low-cost molecular marker panels. Recent studies have shown that a relatively small number of highly informative SNPs can achieve accurate breed classification. For instance, Dimauro et al. selected 48 SNPs from a 50K SNP chip to accurately distinguish three Italian cattle breeds [57], and Liang et al. achieved 98.8% classification accuracy for eight buffalo breeds using mRMR-selected SNPs combined with SVM [58]. In this study, 134 SNPs selected through the combined use of *F*_ST_ and KNN achieved an accuracy of 95.1%, enabling accurate classification of most Chinese dairy goat breeds. These results provide an important basis for developing cost-effective molecular identification tools.

Despite the overall high classification performance, misclassifications were observed among genetically closely related breeds. This may be attributable to low genetic differentiation and historical gene flow associated with breeding stock exchange or historical hybridization. These results suggest that distinguishing highly similar populations remains challenging when relying on a limited number of markers. In addition, differences in sample size among breeds may also influence classification performance to some extent. Therefore, future studies should incorporate larger and more balanced population datasets and integrate multiple feature selection strategies and modeling approaches to further improve classification accuracy and robustness.

### Environmental adaptation of the Chinese dairy goat

During long-term artificial breeding, Chinese dairy goats have developed diverse populations adapted to different climatic environments. Based on gene–environment association analysis, this study identified candidate genes and adaptive signatures associated with climatic adaptation in Chinese dairy goats across different regions. In previous environmental adaptability studies of Hu sheep [45], Toggenburg goats [12], cashmere goats, and Xinjiang sheep [59], genes associated with energy metabolism, stress response, reproduction, and immune regulation have been identified. In this study, we similarly identified candidate genes involved in these functional categories, further supporting the role of these biological processes in climatic adaptation of ruminants. For example, *GPAM*, *MGLL*, *SLC5A8*, *HACD1*, and *HACD2* are involved in key steps of lipid metabolism, including triglyceride synthesis, hydrolysis, fatty acid transport, and elongation, which may facilitate dynamic allocation of energy in high temperature or dry heat environments [60–63]. *ADCY5* catalyzes the conversion of ATP to cAMP and participates in energy metabolism [64] and may also affect seasonal reproduction by regulating steroid hormone synthesis [65]. *BDKRB1* and *BDKRB2* are involved in responses to heat, cold, and oxidative stress, contributing to thermoregulation in dairy cattle and chickens [66–68]. *AGT* is a key component of the renin angiotensin aldosterone system and plays a central role in blood pressure regulation and maintenance of water salt balance [69]. In addition, *GRIA2* may indirectly influence reproductive performance through modulation of excitatory neural signaling and the hypothalamic–pituitary–gonadal axis [70, 71], and *GRM8* and *SMAD3* have been associated with heat tolerance in Holstein cattle [72], indicating multiple physiological and neuroendocrine mechanisms underlying climatic adaptation in dairy goats. High temperature or extremely low environmental temperature can increase the risk of inflammation and oxidative stress in animals [73]. *IL1RL1* and *TRIL* are involved in inflammatory signaling and innate immune regulation, and have been associated with immune traits in Iranian sheep [74, 75]. *HDAC10* is a histone deacetylase involved in melanogenesis and epigenetic regulation of inflammation and stress responses and may contribute to adaptive plasticity under harsh environmental conditions such as high temperature and drought [76]. Interestingly, a missense mutation in *HDAC10* (chr5:118557145 A > G) was identified, with allelic frequencies significantly differing between southern and northern dairy goat populations, suggesting a potential role in the environmental adaptation of Chinese dairy goats under conditions such as high temperature and drought.

Beyond metabolic and stress response pathways, developmental regulators may also contribute to long-term environmental adaptation. In particular, we detected the *HOXD* gene cluster (*HOXD8–13*), which plays essential roles in embryonic forelimb and hindlimb patterning [77] and has been identified as a key regulator of skeletal development in duck embryos [78]. This suggests that long-term environmental pressures may also influence developmental processes related to morphological adaptation. Collectively, these findings indicate that climatic and geographic environmental factors have played important roles in shaping the adaptive genetic structure of Chinese dairy goats. Nevertheless, environmental adaptation is a complex process influenced by multiple ecological factors. Future studies integrating finer-scale ecological stratification, altitude gradients, and high-resolution climatic variables may further improve the understanding of genotype–environment interactions and local adaptation mechanisms in dairy goat populations.

### Genetic basis and candidate gene analysis of horn type in dairy goats

Through long-term breeding, Chinese dairy goats have undergone differentiation into horned and polled types, and polled individuals are associated with improved animal welfare and greater suitability for modern intensive management systems. However, the genetic basis of horn variation in Chinese dairy goats remains poorly understood. Previous studies have reported that the polled phenotype in goats is associated with an 11.7 kb deletion near 129 Mb on chr1 and an approximately 480 kb insertion in the 150 Mb region [79, 80]. In this study, significant selective signals and GWAS association peaks were identified within the intervals 128.87–129.46 Mb and 149.79–150.14 Mb on chr1. These regions are highly consistent with previously reported polled candidate regions in Jintang Black goats [81] and Xinong Saanen dairy goats [21], and positional differences may arise from variations in reference genome versions. Within these regions, several genes potentially involved in horn development were identified. Among them, *FOXL2* is associated with polled intersex syndrome, and may indirectly influence horn bud formation through regulation of mesenchymal stem cell differentiation [82]. *MRPS22* is involved in reproduction and ovarian development and has been linked to intersexual conditions in goats [21]. *KCNJ15* is associated with horn growth and horn development in Chengde polled goats [83]. *ERG* is involved in angiogenesis, hematopoiesis, and cartilage development, and has been associated with osteoblast-related gene expression and structural traits in cattle, suggesting a potential role in horn traits in Xinong Saanen dairy goats [84, 85]. Notably, a missense variant in *ERG* (chr1:149921788 C > G) was identified and showed a strong association with the polled phenotype. The variant was predicted to affect protein structure and regulatory activity, suggesting that it is a candidate locus associated with horn development in dairy goats. The CC genotype was consistently observed in horned goat populations, whereas the homozygous mutant GG genotype was not detected in any sampled population, including individuals affected by polled intersex syndrome.

Collectively, several additional candidate genes identified within the selective regions may contribute to horn morphogenesis through roles in craniofacial patterning, osteogenic differentiation, and dermal-epidermal signaling. *RXFP2* regulates horn bud formation and has been confirmed as a causal gene for polled phenotypes in sheep and cattle [86]. *FOXO1* has been reported to inhibit osteoprogenitor proliferation and bone formation [87]. *ALX1* regulates mesenchymal cell migration and osteogenesis and is associated with craniofacial development and horn morphology [88]. *SHH* is highly expressed in horn buds and promotes horn germ growth and morphogenesis via signaling pathways [89]. *CHD7* regulates neural crest cell differentiation and contributes to horn bud mesenchyme development [90]. The identification of these genes suggests that horn development in dairy goats is likely shaped by coordinated interactions among multiple developmental pathways.

### Analysis of candidate genes for coat color traits in dairy goats

Coat color is an important phenotypic trait in goats, shaped by natural and artificial selection, and serves as a key characteristic for distinguishing between different breeds. During long-term selective breeding, goats have developed diverse coat color variations, while Chinese dairy goat populations have gradually formed a predominantly white coat color trait. Combined selective sweep and GWAS analyses identified several candidate genes associated with coat color variation in Chinese dairy goats, including *ASIP*, *AHCY*, *ITCH*, *KIT*, *MITF*, and *TYR*. These genes were enriched in melanogenesis, Hedgehog signaling, TGF-β signaling, cellular senescence, and cell cycle pathways, indicating that coat color variation may involve multiple conserved pigmentation-related pathways.

Among these, *ASIP* regulates pheomelanin synthesis through its antagonistic interaction with melanocortin receptor 1 (*MC1R*), influencing red, yellow, and tan pigmentation. *ASIP* and *AHCY* have been associated with black coat color variation in goat populations from southwestern China, whereas *ITCH* is linked to white coat expression [91]. In addition, *TYR* and *TYRP1* encode key enzymes in melanin biosynthesis, while *KIT* is involved in melanocyte migration and localization and has been associated with black coat formation [92]. *MITF* is a key regulator of melanocyte development, proliferation, and survival and has been implicated in pigmentation variation across multiple species, including buffalo, mice, and Beijing ducks [19, 93, 94]. Notably, a missense variant in *MITF* (chr22:31686241 T > C) was significantly associated with white and black coat color phenotypes, indicating its potential role in pigmentation differentiation in dairy goats. However, coat color is likely influenced by interactions among multiple regulatory loci rather than by a single causal mutation. These findings suggest that the predominance of white coat color in Chinese dairy goats may reflect long-term artificial selection acting cumulatively on conserved melanogenic regulatory pathways, while breed-specific genetic variation may further contribute to phenotypic diversification among dairy goat populations.

## Conclusion

This study systematically characterized the population genomics of Chinese dairy goats, revealing their genetic structure, genetic diversity, and gene flow from introduced breeds. Candidate genes associated with environmental adaptation, horn type, and coat color were identified, together with three missense variants potentially associated with adaptation and phenotypic specificity in dairy goats. In addition, breed classification SNP markers were screened using machine learning approaches, and an optimal classification panel for Chinese dairy goat breeds was constructed. Collectively, these findings provide an important scientific basis for precision breeding, conservation, and sustainable utilization of Chinese dairy goat genetic resources.

## Supplementary Information

Additional file 1: Table S1. 270 goats Sample information. Table S2. Proportion of annotated SNPs in different genomic regions. Table S3. Cross-validation error values from five replicate ADMIXTURE runs for each K. Table S4. Number of small ROH segments in four populations. Table S5. Inbreeding coefficients of four populations based on small segments of ROH. Table S6. Performance evaluation of five machine learning models across different SNP marker sizes based on 10 repeated tests. Table S7. KNN classification performance evaluated using cumulative SNP subsets ranked by importance. Table S8. Ranking and genomic positions of the 134 selected SNP markers. Table S9. 149 candidate genes annotated from the 134-SNP panel. Table S10. Significant *P*-values and Z–scores for BIO3, BIO8, and BIO9 environmental variables. Table S11. Gene annotation of significant SNPs associated with BIO3, BIO8, and BIO9. Table S12. Candidate genomic regions associated with horn type within the top 5% of *F*_ST_ values. Table S13. Candidate genomic regions associated with horn type within the top 5% of θπ values. Table S14. Candidate genomic regions associated with horn type within the top 5% of ZHp values. Table S15. Candidate genes for horn type identified in overlapping top 5% regions of *F*_ST_, θπ, and ZHp. Table S16. Significant GWAS loci associated with horn type. Table S17. Candidate genes annotated from significant GWAS loci with horn type. Table S18. Candidate genomic regions associated with coat color within the top 5% of *F*_ST_ values. Table S19. Candidate genomic regions associated with coat color within the top 5% of θπ values. Table S20. Candidate genomic regions associated with coat color within the top 5% of ZHp values. Table S21. Candidate genes for coat color identified in overlapping top 5% regions detected by at least two of the *F*_ST_, θπ, and ZHp methods. Table S22. Significantly enriched KEGG pathways of genes associated with coat color identified from *F*_ST_, θπ, and ZHp. Table S23. Significant GWAS loci associated with coat color. Table S24. Candidate genes annotated from significant GWAS loci with coat color.

Additional file 2: Fig. S1 Principal component analysisof Chinese dairy goat populations. A PC1 vs. PC2; B PC1 vs. PC3. Fig. S2 Population structure of 16 populations across K = 2–15. Fig. S3 Model fit and inference of migration edges in TreeMix analysis. Fig. S4 Residual covariance matrix from TreeMix analysis. Fig. S5 Historical changes in effective population size (*Ne*) inferred by SMC++ for different goat populations. Fig. S6 Genomic inbreeding coefficients (*F*_ROH_) estimated from ROH of different minimum lengths. Fig. S7 Nucleotide diversity (π) across different goat populations. Fig. S8 Distribution of genomic inbreeding coefficients (*F*_ROH_) estimated from runs of homozygosity (ROH) across goat populations. Fig. S9 Confusion matrix of the KNN model based on 800 SNPs across 10 independent repetitions. Fig. S10 Breed-level recall matrices for the 800-SNP, 351-core-SNP, and 134-SNP panels. Fig. S11 Confusion matrix of 10-fold predictions based on 134 SNP markers. Fig. S12 Recall matrix of 10-fold predictions based on 134 SNP markers. Fig. S13 Heat map of correlation analysis of 19 environmental factors. Fig. S14 Correlation heat map of three environmental factors: BIO3, BIO8, and BIO9. Fig. S15 Distribution of BIO3, BIO8 and BIO9 in Chinese dairy goat population. Fig. S16 RDA projection plot on RDA1 and RDA2. Fig. S17 Analysis of selection signals for horned and polled goats. A *F*_ST_; B θπ; C ZHp. Fig. S18 KEGG enrichment analysis of candidate genes associated with horn type under selection signals. Fig. S19 QQ plots of GWAS for horned and polled goats. Fig. S20 −log_10_(*P*-value) scatter plot (top) and linkage disequilibrium (LD) block analysis (bottom) of the 128.8-129.5 Mb. Fig. S21 Comparison of allele frequencies (C and G) between horned and polled groups (^***^P < 0.001). Fig. S22 Distribution of allele frequencies at the chr1:149921788 C>G site among global goat populations. Fig. S23 Phylogenetic tree of ERG protein sequences from 25 species. Fig. S24 Analysis of selection signals for coat color in dairy goats based on *F*_ST_. Fig. S25 Analysis of selection signals for coat color in dairy goats based on θπ. Fig. S26 QQ plots of GWAS for coat color in goats.

## Data Availability

The publicly available whole-genome sequencing data used in this study can be accessed under the sample accession numbers listed in Additional file 1: Table S1. Other genomic data generated in this study are not publicly available but can be obtained from the corresponding author upon reasonable request.

## Abbreviations

AUC: Area under the curve
chr: Chromosome
DT: Decision tree
*F*_ROH_: Genomic inbreeding coefficient based on runs of homozygosity
*F*_ST_: Fixation index
GWAS: Genome-wide association study
κ: Cohen’s kappa coefficient
KEGG: Kyoto Encyclopedia of Genes and Genomes
KNN: K-nearest neighbors
LD: Linkage disequilibrium
LFMM: Latent Factor Mixed Model
*Ne*: Effective population size
NJ: Neighbor-Joining
θπ: Nucleotide diversity estimator
PCA: Principal component analysis
RF: Random forest
ROH: Runs of homozygosity
SNP: Single nucleotide polymorphism
SVM: Support vector machine
VIF: Variance inflation factor
XGBoost: Extreme gradient boosting
ZHp: Z-transformed haplotype homozygosity
