# Supplementary material for: Integrating landscape genomics and machine learning to analyze the genetic structure and specific molecular markers of Chinese dairy goats (Capra hircus)
Source: J Anim Sci Biotechnol. 2026 Aug 6;17:155. doi: 10.1186/s40104-026-01473-0 (PMC13445786; doi:10.1186/s40104-026-01473-0)
Supplement: Supplementary file 1 [file 40104_2026_1473_MOESM2_ESM.docx]

**Additional file 2**

**Integrating landscape genomics and machine learning to analyze the genetic structure and specific molecular markers of Chinese dairy goats (*Capra hircus*)**

Jinke Xiong, Mingyu Shang, Jingjing Bao, Tianyi Liu, Peifu Yang, Xintian Sun, Yuting Jiang, Li Zhang*

State Key Laboratory of Animal Biotech Breeding, Institute of Animal Science, Chinese Academy of Agricultural Sciences (CAAS), Beijing, China, 100193.

* Correspondence:

Li Zhang

zhangli07@caas.cn

**Supplementary information includes:**

Fig. S1 Principal component analysis (PCA) of Chinese dairy goat populations. (A) PC1 vs PC2; (B) PC1 vs PC3.

Fig. S2 Population structure of 16 populations across K = 2-15.

Fig. S3 Model fit and inference of migration edges in TreeMix analysis.

Fig. S4 Residual covariance matrix from TreeMix analysis.

Fig. S5 Historical changes in effective population size (*Ne*) inferred by SMC++ for different goat populations.

Fig. S6 Genomic inbreeding coefficients (*F*_ROH_) estimated from ROH of different minimum lengths.

Fig. S7 Nucleotide diversity (π) across different goat populations.

Fig. S8 Distribution of genomic inbreeding coefficients (*F*_ROH_) estimated from runs of homozygosity (ROH) across goat populations.

Fig. S9 Confusion matrix of the KNN model based on 800 SNPs across 10 independent repetitions.

Fig. S10 Breed-level recall matrices for the 800-SNP, 351-core-SNP, and 134-SNP panels.

Fig. S11. Confusion matrix of 10-fold predictions based on 134 SNP markers.

Fig. S12. Recall matrix of 10-fold predictions based on 134 SNP markers.

Fig. S13 Heat map of correlation analysis of 19 environmental factors.

Fig. S14 Correlation heat map of three environmental factors: BIO3, BIO8, and BIO9.

Fig. S15 Distribution of BIO3, BIO8 and BIO9 in Chinese dairy goat population.

Fig. S16 RDA projection plot on RDA1 and RDA2.

Fig. S17 Analysis of selection signals for horned and polled goats. (A) *F*_ST_; (B) θπ; (C) ZHp.

Fig. S18 KEGG enrichment analysis of candidate genes associated with horn type under selection signals.

Fig. S19 QQ plots of GWAS for horned and polled goats.

Fig. S20 –log_10_(*P*-value) scatter plot (top) and linkage disequilibrium (LD) block analysis (bottom) of the 128.8-129.5 Mb.

Fig. S21 Comparison of allele frequencies (C and G) between horned and polled groups (*** *P* < 0.001).

Fig. S22 Distribution of allele frequencies at the chr1:149921788 C>G site among global goat populations.

Fig. S23 Phylogenetic tree of ERG protein sequences from 25 species.

Fig. S24. Analysis of selection signals for coat color in dairy goats based on *F*_ST_.

Fig. S25. Analysis of selection signals for coat color in dairy goats based on θπ.

Fig. S26 QQ plots of GWAS for coat color in goats.

**Fig. S1** Principal component analysis (PCA) of Chinese dairy goat populations. (A) PC1 vs PC2; (B) PC1 vs PC3.

**Fig. S2** Population structure of 16 populations across K = 2-15.

**Fig. S3** Model fit and inference of migration edges in TreeMix analysis.

The top panel shows the mean log-likelihood (black circles, ±SD) and the percentage of variance explained (red dots) as a function of the number of migration edges (m). The dotted line indicates the 99.8% variance explained threshold. The bottom panel displays the change in log-likelihood (Δm) between successive models. The selected migration model was determined based on the Δm statistic and the proportion of variance explained across models.

**Fig. S4** Residual covariance matrix from TreeMix analysis.

The heatmap shows the residuals from the TreeMix model, representing the difference between the observed and expected covariance of allele frequencies among populations. Positive residuals (red) indicate that pairs of populations share more alleles than expected under the model, suggesting potential unmodeled gene flow, while negative residuals (blue) indicate the opposite. The color scale denotes residuals in standard error (SE) units.

**Fig. S5** Historical changes in effective population size (*Ne*) inferred by SMC++ for different goat populations.

**Fig. S6** Genomic inbreeding coefficients (*F*_ROH_) estimated from ROH of different minimum lengths.

Violin plots show the distribution of F*_ROH_* values across goat populations, calculated from runs of homozygosity (ROH) with minimum lengths of 0.1 Mb (A), 0.2 Mb (B), 0.3 Mb (C), and 0.4 Mb (D), respectively. Wider sections of the violins indicate higher data density.

**Fig. S7** Nucleotide diversity (π) across different goat populations.

**Fig. S8** Distribution of genomic inbreeding coefficients (*F*_ROH_) estimated from runs of homozygosity (ROH) across goat populations.

**Fig. S9** Confusion matrix of the KNN model based on 800 SNPs across 10 independent repetitions.

**Fig. S10** Breed-level recall matrices for the 800-SNP, 351-core-SNP, and 134-SNP panels.

**Fig. S11.** Confusion matrix of 10-fold predictions based on 134 SNP markers.

**Fig. S12.** Recall matrix of 10-fold predictions based on 134 SNP markers.

**Fig. S13** Heat map of correlation analysis of 19 environmental factors

**Fig. S14** Correlation heat map of three environmental factors: BIO3, BIO8, and BIO9.

**Fig. S15** Distribution of BIO3, BIO8 and BIO9 in Chinese dairy goat population.

**Fig. S16** RDA projection plot on RDA1 and RDA2.

**Fig. S17** Analysis of selection signals for horned and polled goats. (A) *F*_ST_; (B) θπ; (C) ZHp.

**Fig. S18** KEGG enrichment analysis of candidate genes associated with horn type under selection signals.

**Fig. S19** QQ plots of GWAS for horned and polled goats.

**Fig. S20**–log_10_(*P*-value) scatter plot (top) and linkage disequilibrium (LD) block analysis (bottom) of the 128.8-129.5 Mb

**Fig. S21** Comparison of allele frequencies (C and G) between horned and polled groups. *** indicates *P* < 0.001.

**Fig. S22** Distribution of allele frequencies at the chr1:149921788 C>G site among global goat populations.

**Fig. S23** Phylogenetic tree of ERG protein sequences from 25 species.

**Fig. S24**. Analysis of selection signals for coat color in dairy goats based on *F*_ST._

**Fig. S25**. Analysis of selection signals for coat color in dairy goats based on θπ.

**Fig. S26** QQ plots of GWAS for coat color goats.
